# Alkoxy Side Chain Engineering in Metal‐Free Covalent Organic Frameworks for Efficient Oxygen Reduction

**DOI:** 10.1002/adma.202501603

**Published:** 2025-07-04

**Authors:** Zhongping Li, Zhaoying Wang, Songlin Zhao, Jeong‐Min Seo, Changqing Li, Yucheng Jin, Siliu Lyu, Jian Li, Feng Tang, Won‐Yeong Kim, Zonghoon Lee, Sang‐Yong Lee, Jong‐Beom Baek

**Affiliations:** ^1^ Key Laboratory of Automobile Materials of MOE and School of Materials Science and Engineering Jilin University Changchun 130012 P. R. China; ^2^ School of Energy & Chemical Engineering Ulsan National Institute of Science and Technology (UNIST) Ulsan 44919 Republic of Korea; ^3^ School of Chemistry and Chemical Engineering Shandong University of Technology Zibo 255036 P.R. China; ^4^ Department of Chemical and Biomolecular Engineering Yonsei University Seoul 03722 Republic of Korea; ^5^ Department of Materials Science and Engineering Ulsan National Institute of Science and Technology (UNIST) Ulsan 44919 Republic of Korea

**Keywords:** alkoxy side chains, covalent organic frameworks, electronic states, hydrophilic skeleton, oxygen reduction reaction (ORR)

## Abstract

Metal‐free covalent organic frameworks (COFs) gain significant attention as catalysts for the oxygen reduction reaction (ORR), a key process in energy conversion technologies like fuel cells and metal–air batteries. While substantial efforts are devoted to unravelling the mechanisms, by which heteroatom‐containing building blocks in linkers, vertices, and linkages, enhance catalytic activity and selectivity, the potential of side‐chain engineering to modulate pore wall surfaces and optimize the catalytic environment remains largely underexplored. This study investigates the role of alkoxy side chains in modulating the properties of COFs to enhance ORR performance. The synthesized COFs have adjustable pore surfaces, integrating triazine rings and alkoxy groups to enhance channel hydrophilicity by modulating interactions with water molecules. Moreover, the alkoxy side chains act as electron donors through p–π conjugation, creating active and tuneable electronic sites, further enhancing hydrophilicity and facilitating efficient catalytic cycles. Notably, COFs with longer alkoxy side chains exhibit superior ORR activity, with a half‐wave potential of 0.77 V, surpassing previously reported metal‐free COFs. Theoretical calculations suggest that this enhancement is because of the stronger binding affinity of water molecules and *OOH intermediates to the carbon atoms adjacent to the alkoxy side chains.

## Graphical abstract

This study explores how alkoxy side chains influence COF properties to enhance oxygen reduction reaction (ORR) performance. Incorporating varying alkoxy groups improves hydrophilicity and modulates the electronic structure via p‐π conjugation. Notably, longer alkoxy side chains facilitate fine‐tuning of the COFs' electronic states, resulting in enhanced ORR activity that outperforms previously reported metal‐free COFs.

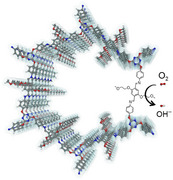

## 1 Introduction

The oxygen reduction reaction (ORR) is a key process in clean energy technologies such as metal–air batteries and fuel cells. As promising alternatives to platinum‐based electrocatalysts for ORR applications, metal‐free carbon‐based materials have gained interest because of their effective ORR activity.^[^ ^1^, ^2^, ^3^ ^]^ However, these conventional carbon materials are limited by an insufficient number of active sites for oxygen adsorption and by the slow activation of ORR intermediates, which hinder their catalytic efficiency. Addressing these challenges requires precise modulation of their electronic properties.^[^ ^4^, ^5^, ^6^ ^]^ Recent advances in defect engineering, heteroatom doping, and high‐temperature pyrolysis have shown their significant potential for enhancing catalytic performance.^[^ ^5^ ^]^ Nevertheless, achieving precise control of active site accessibility within an optimized catalytic environment remains a major challenge, underscoring the need for further development of advanced, scalable electrocatalysts.^[^ ^6^, ^7^ ^]^

Covalent organic frameworks (COFs) are a versatile class of porous polymers composed of covalently bonded building blocks.^[^ ^8^, ^9^, ^10^, ^11^, ^12^, ^13^, ^14^, ^15^ ^]^ COFs can be engineered to exhibit a wide range of dimensional topologies, pore shapes, and sizes.^[^ ^16^, ^17^, ^18^, ^19^, ^20^, ^21^ ^]^ This versatility makes them widely customizable for diverse applications. In addition to their structural flexibility, COFs also exhibit exceptional functional tunability, making them suitable for applications such as gas adsorption, photocatalysis, and energy storage and conversion.^[^ ^22^, ^23^, ^24^, ^25^, ^26^, ^27^, ^28^, ^29^, ^30^, ^31^, ^32^, ^33^, ^34^, ^35^, ^36^, ^37^, ^38^, ^39^, ^40^ ^]^ Recently, COFs have gained attention as a promising electrochemical platform because of their combination of structural stability, high efficiency, and catalytic activity, giving them substantial potential as ORR catalysts.^[^ ^16^, ^40^, ^41^, ^42^ ^]^

COFs are characterized by well‐defined, 2D polygonal skeletons that integrate electroactive sites in the organic linkers, the vertices, or the linkages embedded within their walls.^[^ ^36^, ^37^, ^40^, ^41^, ^42^, ^43^, ^44^, ^45^ ^]^ Incorporating heteroatoms, such as bithiophene, thiophene, benzotrithiophene, benzothiadiazole, and azo units, into these linkers, the vertices, or the linkages has been shown to enhance ORR performance by stabilizing reaction intermediates and promoting efficient electron transfer.^[^ ^40^, ^41^, ^42^, ^43^, ^44^, ^45^, ^46^ ^]^ However, despite these advances, the electronic activity at the pore wall surfaces of COFs remains constrained, limiting their full catalytic potential. While the pore walls contribute to the extended conjugated skeleton and modulate electronic properties, they also form π‐stacked columns that regulate host–guest molecular interactions and influence the hydrophilicity and hydrophobicity of the material.^[^ ^8^, ^9^, ^47^, ^48^ ^]^ Overcoming the intrinsic electrochemical limitations of the pore wall surfaces is critical to unlock innovative design strategies and develop highly active COF‐based electrocatalysts.

**Figure 1:**
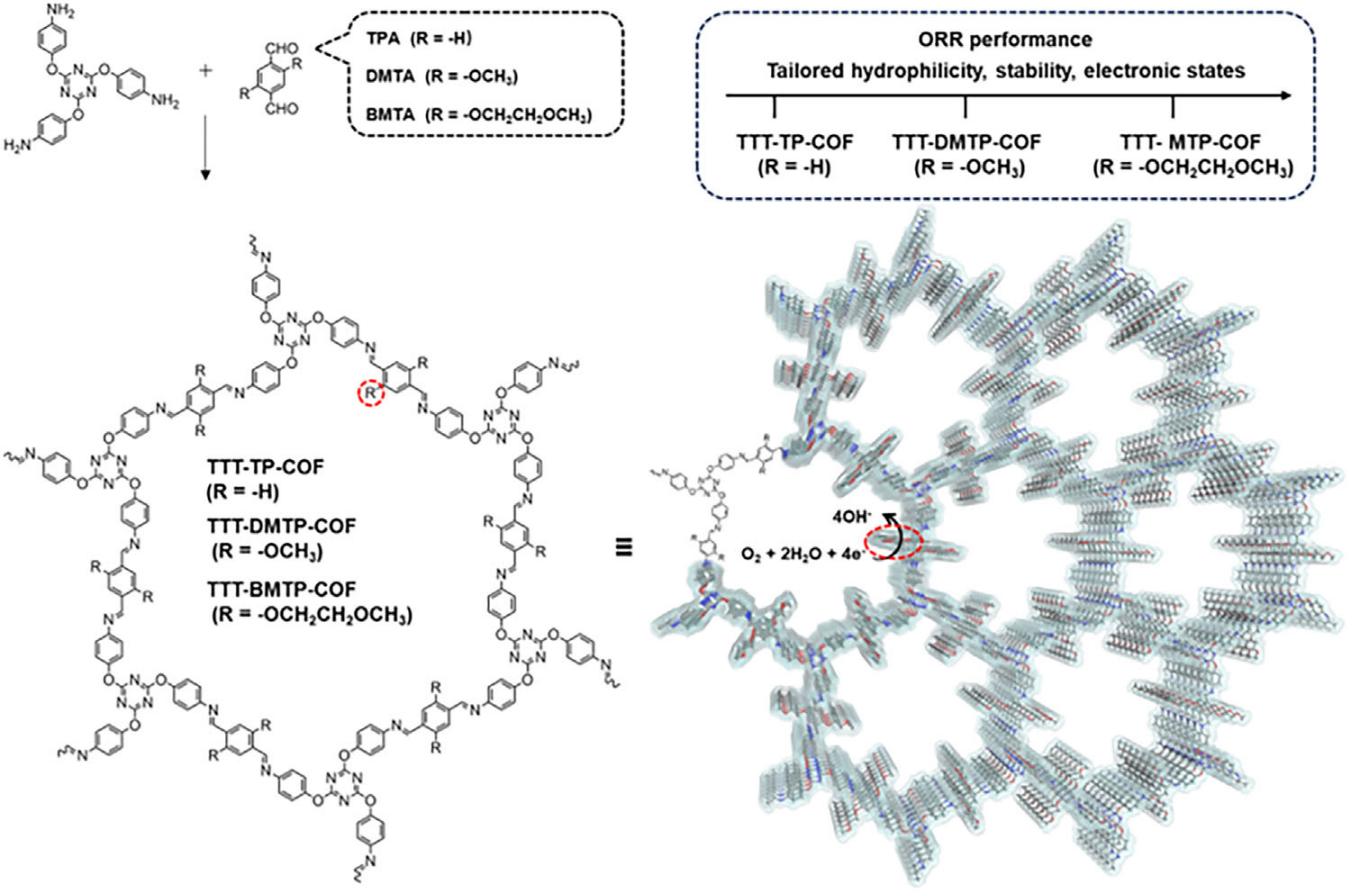
Schematic representation of the design strategy for side‐chain‐modified COFs as electrocatalysts for the ORR process.

To address these challenges, we synthesized imine‐linked COFs with tunable pore surfaces and incorporated triazine rings and alkoxy side groups to enhance hydrophilicity within the channels by modulating interactions with water molecules (**Figure** **1**). Additionally, the alkoxy side chains, acting as electron donors through p–π conjugation, served as tunable electronic sites, further enhancing hydrophilicity and facilitating efficient catalytic cycles. These modifications successfully optimized critical properties, including tunable pore surface characteristics, and enhanced hydrophilicity, improved stability, and tailored electronic states at the catalytic centers, enabling precise control over both hydrophilicity and electrochemical activity within the channels. Under optimal conditions, the COFs modified with longer alkoxy side chains exhibited the highest ORR activity, with a half‐wave potential of 0.77 V and a mass activity of 17.6 A g^−1^. Theoretical calculations attribute this enhancement to the increased binding affinity of water molecules and *OOH intermediates to the carbon atoms adjacent to the alkoxy side chains, which facilitates the proton‐coupled electron transfer steps critical for efficient ORR. In addition, the longer alkoxy side chains help to stabilize reaction intermediates and reduce the energy barriers associated with oxygen and water conversion, further improving catalytic performance.

## 2 Results and Discussion

COFs with different side chains were meticulously designed and synthesized via reversible Schiff‐base condensation between 4,4′,4″‐((1,3,5‐triazine‐2,4,6‐triyl)tris(oxy))trianiline (TTTOA) and aromatic aldehydes, namely terephthalaldehyde (TP), 2,5‐dimethoxyterephthalaldehyde (DMTA), or 2,5‐bis(2‐methoxyethoxy)terephthalaldehyde (BMTA), respectively, to yield TTT‐TP‐COF, TTT‐DMTP‐COF, and TTT‐BMTP‐COF (Figure 1). The chemical structures of these three COFs were confirmed via Fourier transform infrared (FT‐IR) spectroscopy. The emergence of new stretching vibration peaks at 1599–1594 cm^−1^ in three COFs indicated the formation of imine linkage (Figure S1, Supporting Information). Additionally, the stretching vibration signatures from 2860 to 3000 cm^−1^ were attributed to alkoxy side groups in TTT‐DMTP‐COF and TTT‐BMTP‐COF. X‐ray photoelectron spectroscopy analyses confirmed the structure details of synthesized COFs, where the carbon (C) 1s, oxygen (O) 1s and nitrogen (N) 1s signals were obviously detected (Figure S2a, Supporting Information), and deconvoluted N 1s spectrum proved the composition of imine linkage within the obtained COF materials (Figure S2b, Supporting Information). Elemental analysis (EA) results showed that the carbon, nitrogen, and hydrogen compositions of the COFs were close to the theoretical values (Table S1, Supporting Information). Furthermore, carbon thirteen (^13^C) magic angle spin solid‐state nuclear magnetic resonance spectroscopy revealed the characteristic carbon peaks of imine linkage at 158.3, 159.2, and 159.1 ppm (Figure S3, Supporting Information). The alkoxy carbons in TTT‐DMTP‐COF and TTT‐BMTP‐COF were observed to be in the range of 53 to 72 ppm. Field‐emission scanning electron microscopy was employed to investigate the morphology, revealing a flaky stacking morphology for TTT‐TP‐COF and a uniform rice grain‐like structure for TTT‐DMTP‐COF and TTT‐BMTP‐COF (Figure S4, Supporting Information). Energy‐dispersive X‐ray mapping images indicated uniform elemental distribution across the three COFs (Figures S5–S7, Supporting Information). Thermogravimetric analysis demonstrated the stability of the three COFs up to 350 °C under a nitrogen atmosphere (Figure S8, Supporting Information).

**Figure 2:**
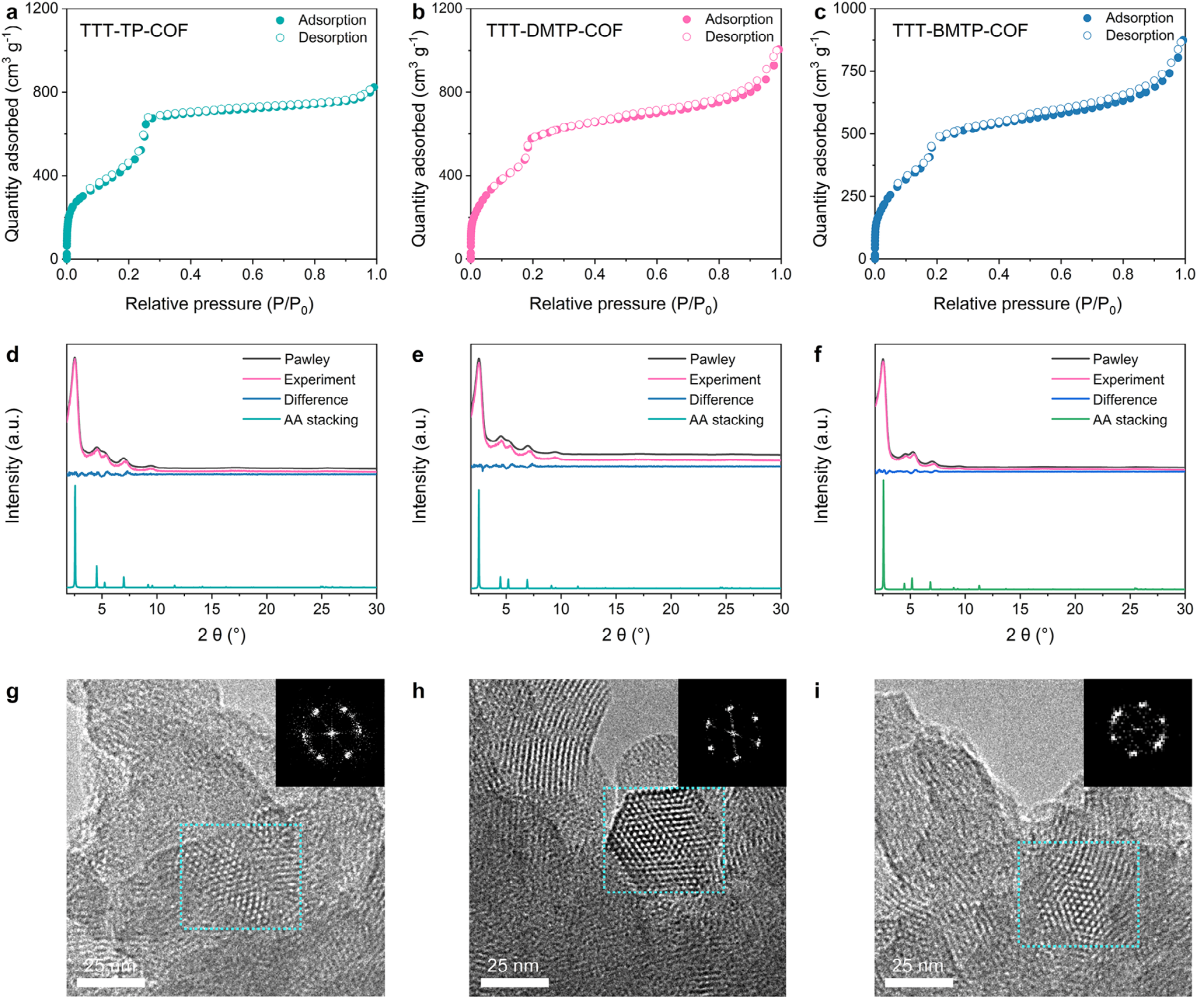
Nitrogen sorption isotherms of a) TTT‐TP‐COF, b) TTT‐DMTP‐COF, and c) TTT‐BMTP‐COF at 77 K. PXRD patterns of d) TTT‐TP‐COF, e) TTT‐DMTP‐COF, and f) TTT‐BMTP‐COF. HR‐TEM images of g) TTT‐TP‐COF, h) TTT‐DMTP‐COF, and i) TTT‐BMTP‐COF. Scale bars: 25 nm. The insets in (g–h) are their corresponding FFT images.

Their porosity and crystallinity, crucial characteristics of the three COFs, were further assessed using nitrogen sorption at 77 K and powder X‐ray diffraction (PXRD) analyses. All three COFs exhibited typical type IV sorption isotherms indicative of mesoporous structures (**Figure** **2**a–c). The high Brunauer–Emmett–Teller surface areas of TTT‐TP‐COF, TTT‐DMTP‐COF, and TTT‐BMTP‐COF were determined to be 1826, 2121, and 1889 cm^2^ g^−1^, respectively. The corresponding pore sizes were calculated to be 3.5 nm for TTT‐TP‐COF (Figure S9a, Supporting Information). When alkoxy side chains are anchored in the channels, the pore sizes slightly decrease to 3.4 and 3.2 nm for TTT‐DMTP‐COF and TTT‐BMTP‐COF, respectively (Figure S9b,c, Supporting Information). The pore size becomes significantly smaller when modified with alkoxy side groups, which is in agreement with the structure reported in the literature.^[^ ^34^, ^35^ ^]^ The PXRD patterns of TTT‐TP‐COF exhibited five peaks with the most intense one at 2.56°, attributed to the (100) facet, along with other peaks at 4.6°, 5.22°, and 7.88° assigned to the (110), (200), and (210) facets, respectively (Figure 2d). Similar peak patterns were observed for TTT‐DMTP‐COF and TTT‐BMTP‐COF (Figure 2e,f). The experimental PXRD patterns of all the BTT‐COFs agreed well with the simulated patterns of Pawley structures. The main difference in the PXRD patterns was observed peaks indicating variations in the AA structure rather than AB stacking (Figure S10, Supporting Information). Detailed information about the crystal structures, including unit cell parameters, is provided in Tables S2–S4 (Supporting Information). High‐resolution transmission electron microscopy (HR‐TEM) confirmed the crystalline pore structures of the three COFs, revealing hexagonal arrangements of white spots that correspond to hexagonally ordered pores. The fast Fourier transform (FFT) images (Figure 2g–i) further indicated the presence of the (100) facet, supporting the ordered pore structure.

**Figure 3:**
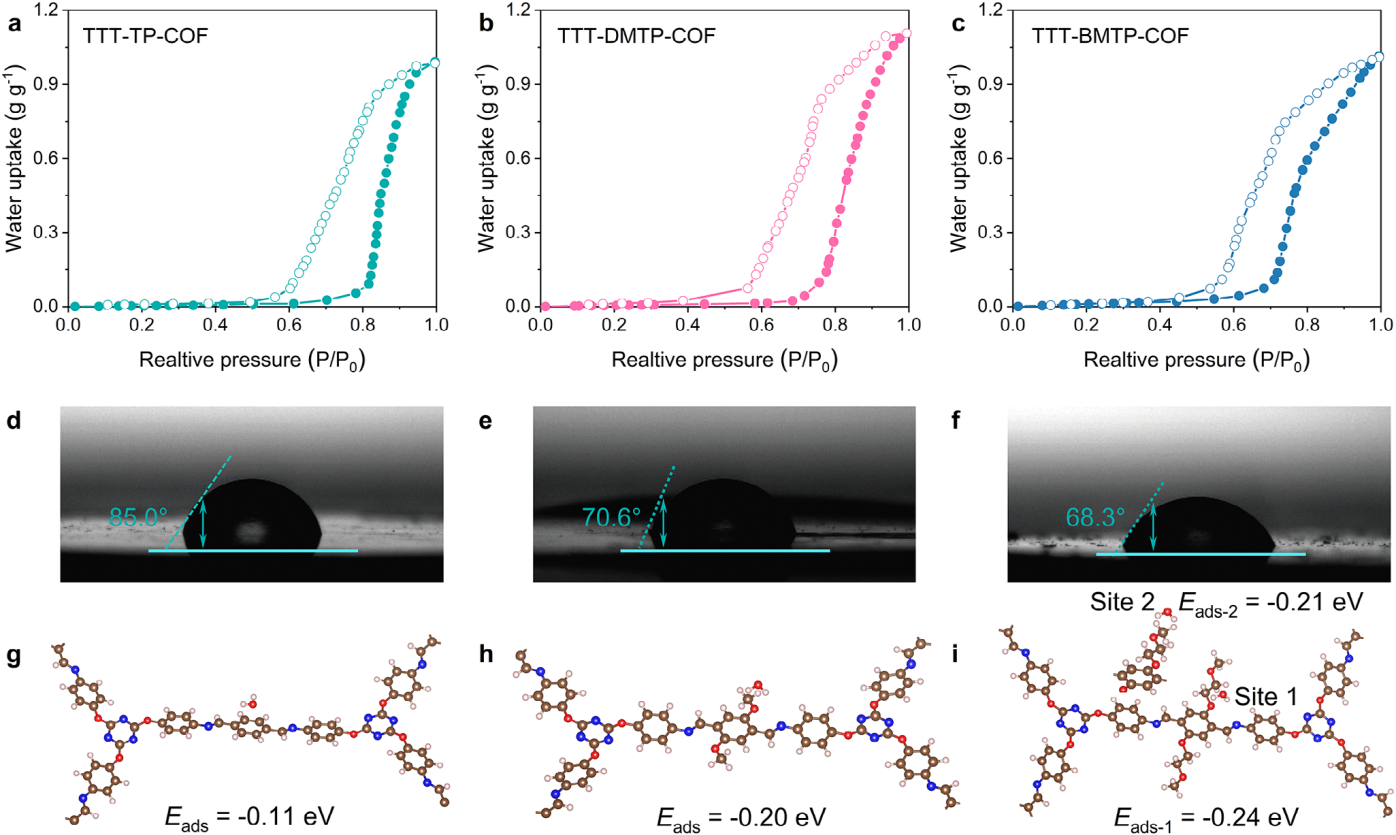
Water sorption isotherms of a) TTT‐TP‐COF, b) TTT‐DMTP‐COF, and c) TTT‐BMTP‐COF at 298 K. Water contact angle (WCA) measurements of d) TTT‐TP‐COF, e) TTT‐DMTP‐COF, and f) TTT‐BMTP‐COF. Water sorption energies of g) TTT‐TP‐COF, h) TTT‐DMTP‐COF, and i) TTT‐BMTP‐COF.

To further assess the stability of these three COFs, they were subjected to immersion in various solutions, including aqueous KOH, aqueous HCl, water, *N*, *N*‐dimethylformamide (DMF), and hexane for 24 h. Both the TTT‐DMTP‐COF and TTT‐BMTP‐COF retained their original skeletal structure and crystalline integrity, as evidenced by the PXRD patterns shown in Figure S11 (Supporting Information), demonstrating better stability than TTT‐TP‐COF. This enhanced stability is attributed to the electron‐donating side chains, which strengthen the COF walls. Specifically, the introduction of methoxy groups to each phenyl edge delocalizes the lone pairs from the oxygen atoms over the central phenyl ring via p–π conjugation, thereby stabilizing the COFs.^[^ ^10^, ^47^, ^48^ ^]^ Water sorption isotherms at 298 K (**Figure** **3**a–c) further revealed that, at 100% relative humidity (RH), the water capacities of the COFs were 0.99, 1.1, and 1.02 g g^−1^, respectively, due to their high porosity. Additionally, TTT‐BMTP‐COF showed a sharp increase in water sorption from 73, 75 to 81% relative humidity (RH), which was attributed to the alkoxy side chains forming hydrogen bonds with water molecules.^[^ ^37^, ^50^ ^]^ This result highlights the crucial role of side‐chain engineering to enhance the hydrophilicity of COF pore surfaces.

These findings were confirmed by water contact angle (WCA) measurements, where TTT‐TP‐COF, TTT‐DMTP‐COF, and TTT‐BMTP‐COF had WCA values of 85°, 70.6°, and 68.3°, respectively (Figure 3d–f). This result confirms the strong binding of water molecules and indicates a high level of hydrophilicity, especially in TTT‐DMTP‐COF and TTT‐BMTP‐COF, due to the presence of alkoxy side chains on their walls.^[^ ^50^, ^51^ ^]^ The advantageous hydrophilic properties of the three COF samples were further verified through density functional theory (DFT) calculations, focusing on the adsorption energy of water onto the COF skeletons. The calculated adsorption energies for TTT‐TP‐COF, TTT‐DMTP‐COF, and TTT‐BMTP‐COF were −0.11, −0.2, and −0.24/−0.21 eV, respectively (Figure 3g–i). The enhanced adsorption energy observed in TTT‐BMTP‐COF was attributed to the presence of alkoxy side chains on the framework walls, which preferentially interacted with water molecules. This interaction increased the number of free water molecules and facilitated a more uniform water flux within the 1D channels.

**Figure 4:**
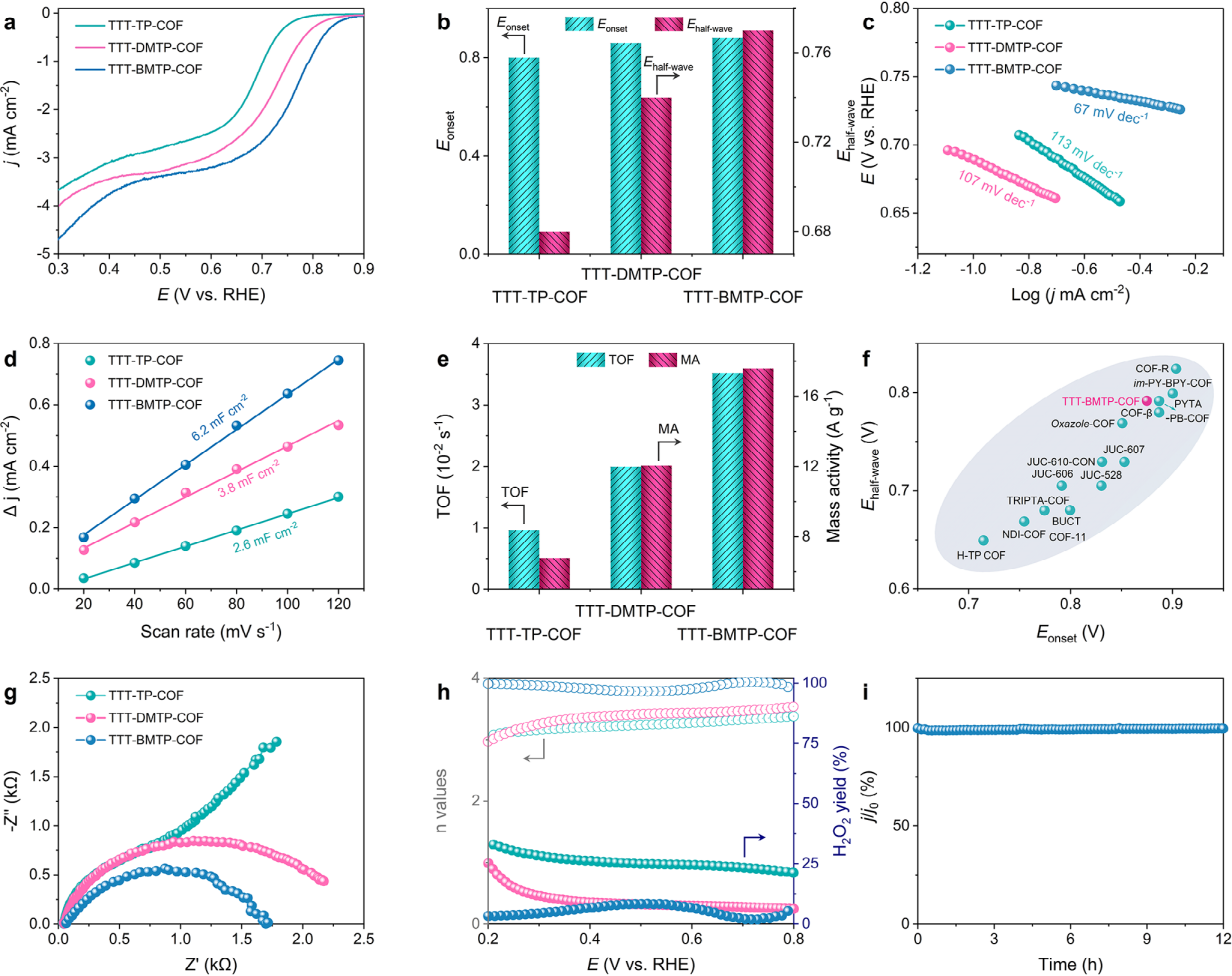
Electrocatalytic activities of TTT‐TP‐COF, TTT‐DMTP‐COF, and TTT‐BMTP‐COF in 0.1 m aq. KOH electrolyte. a) LSV curves at 1600 rpm, b) Comparison of onset and half‐wave potentials, c) Tafel plots, d) *C*_dl_ values, e) TOF values and mass activities, f) Onset and half‐wave potential distributions of reported COF catalysts and TTT‐BMTP‐COF. g) Electrochemical impedance spectroscopy curves, h) Electron transfer number and H_2_O_2_ yield plots calculated from the RRDE measurements, i) Long‐time stability of TTT‐BMTP‐COF.

Given the unique structural merits of excellent crystallinity, stability, and hydrophilicity within the as‐formed COFs, as a proof of concept, the electrocatalytic performance of the COFs toward the ORR was investigated using rotating disk electrode (RDE) measurements in O_2_‐saturated 0.1 m aq. KOH. The linear sweep voltammetry (LSV) test suggested that the TTT‐BMTP‐COF has a more positive half‐wave potential (*E*_1/2_) of 0.77 V versus RHE, compared with TTT‐TP‐COF (0.74 V) and TTT‐DMTP‐COF (0.68 V) (**Figure** **4**a,b; Figure S12, Supporting Information). The Tafel plot (Figure 4c), limiting current density (*j*_L_) and kinetic current density (*j*_K_) graph (Figure S13, Supporting Information) derived from the LSV curves further confirmed the TTT‐BMTP‐COF had better ORR kinetics. It exhibited a smaller Tafel slope of 67 mV dec^−1^, higher *j*_L_ and *j*_K_ value of 3.4 mA cm⁻^2^ and 12.4 mA cm⁻^2^. Cyclic voltammograms (CV) conducted at a non‐Faradic potential range revealed that abundant active sites were available at the solid/liquid interface of TTT‐BMTP‐COF (Figure 4d; Figure S14, Supporting Information). The *C*_dl_ values of TTT‐TP‐COF, TTT‐DMTP‐COF, and TTT‐BMTP‐COF were 2.6, 3.8, and 6.2 mF cm^−2^, respectively, proving the side chain modulation of COFs could substantially increase the active site density for enhanced ORR activity. Although the *C*_dl_‐normalized current density (Figure S15, Supporting Information) of TTT‐BMTP‐COF was comparable to or slightly lower than that of TTT‐TP‐COF at certain potentials (0.7 and 0.6 V vs RHE), several factors should be considered when interpreting intrinsic activity. First, TTT‐BMTP‐COF exhibited significantly lower Tafel slopes and higher charge transfer rates, suggesting more favorable reaction kinetics. Second, the extended conjugation in BMTP units likely maybe enhance charge delocalization, facilitating more efficient electron transport. Last, differences in surface hydrophilicity and porosity might also contribute to improved overall performance, even if normalized *C*_dl_ values are similar. Therefore, the intrinsic activity difference was multifactorial and could not be solely captured by *C*_dl_ normalization.

Turnover of frequency (TOF) and mass activity (MA) calculated at 0.7 V versus RHE were used to reveal the intrinsic activity of the catalysts. As shown in Figure 4e, TTT‐BMTP‐COF had TOF and MA values of 0.035 s^−1^ and 17.6 A g^−1^, respectively, surpassing those of TTT‐DMTP‐COF (0.02 s^−1^ and 12 A g^−1^), TTT‐TP‐COF (0.009 s^−1^ and 6.8 A g^−1^). These results demonstrate that TTT‐BMTP‐COF maintained a higher active site utilization efficiency. The increased hydrophilicity resulting from the electron‐donating effect of the alkoxy side chains on the framework walls simultaneously contributed to favourable electronic properties and interfacial behaviours. By virtue of those features, the as‐constructed TTT‐BMTP‐COF demonstrated catalytic activity competitive with or even superior to the tested COFs in this work and other reported metal‐free COFs (Figure 4f).^[^ ^41^, ^42^, ^43^, ^44^, ^45^, ^46^, ^49^, ^50^, ^51^, ^52^, ^53^, ^54^, ^55^, ^56^, ^57^ ^]^

Further, electrochemical impedance spectroscopy proved that the TTT‐BMTP‐COF possessed the fastest electron transport capability within the electrolyte/catalyst interface. The relatively smaller diameter of the semicircle in the plot signifies the lower resistance of the interfacial kinetic pathway on the surface of TTT‐BMTP‐COF (Figure 4g), which was consistent with their advantageous hydrophilic properties (Figure 3). Electron transfer number and H_2_O_2_ yield plots calculated from the RRDE measurement (Figure 4h) validated the near‐complete four‐electron reduction process of TTT‐BMTP‐COF toward ORR. Over a wide potential range of 0.2–0.8 V versus RHE, TTT‐BMTP‐COF possessed an electron transfer number higher than 3.8 and a low H_2_O_2_ yield at ≈8%. Those metrics obviously exceeded the performance of TTT‐TP‐COF (3.1 and 21%) and TTT‐DMTP‐COF (3.2 and 11%), confirming its higher selectivity. The long‐term stability study (Figure 4i) underscores the robust structural integrity of TTT‐BMTP‐COF, and its huge potential as a durable and reliable catalyst for next‐generation energy storage devices.

**Figure 5:**
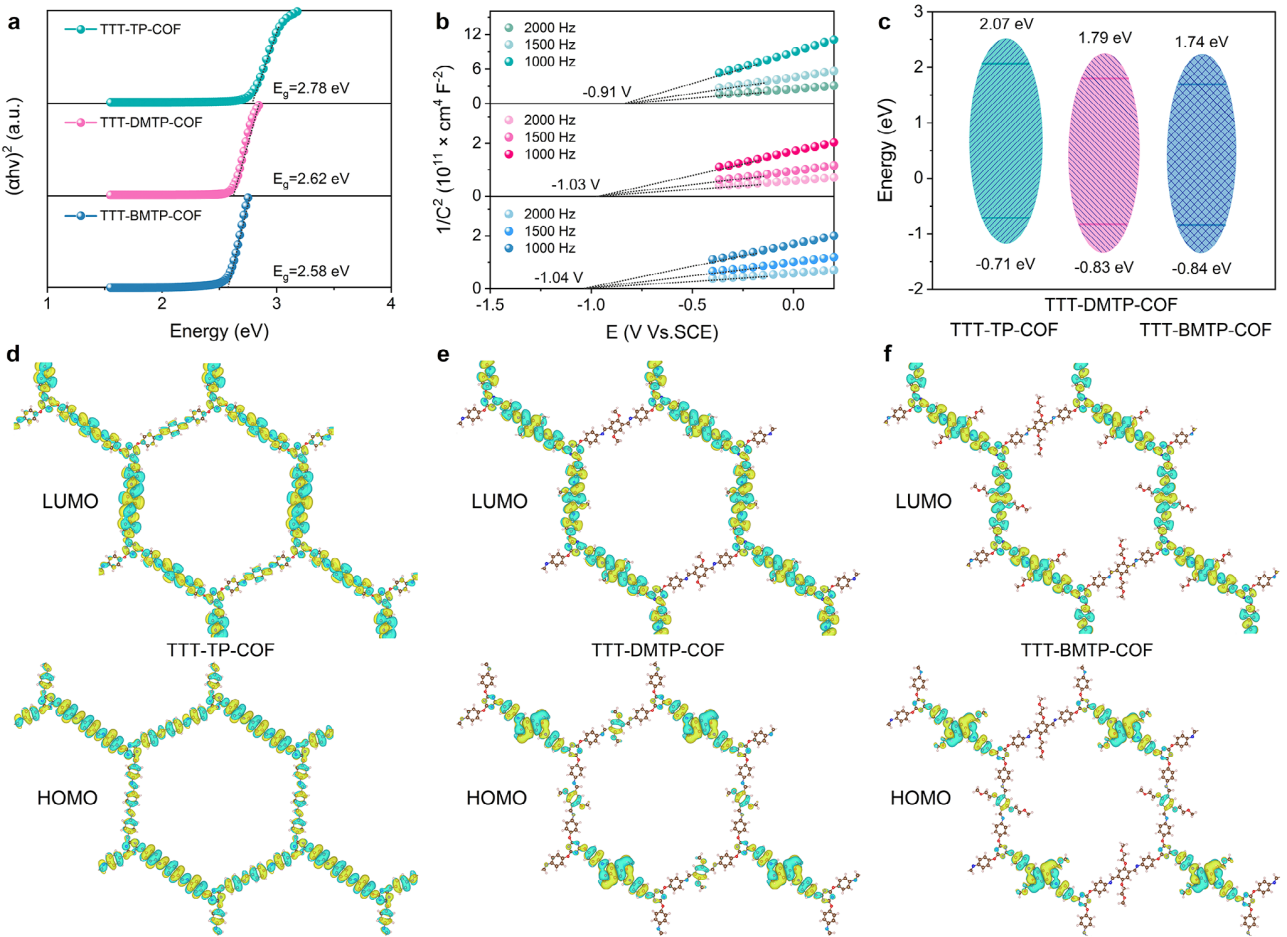
a) Tauc plots by electronic absorption spectra, b) Mott–Schottky curves, and c) schematic energy band structures of COFs. Depictions of the LUMOs and HOMOs for d) TTT‐TP‐COF, e) TTT‐DMTP‐COF, and f) TTT‐BMTP‐COF models.

To investigate the distinct catalytic properties of the COFs, both experimental analyses and DFT calculations were carried out. The electronic structures of the three COFs were examined using UV–vis spectroscopy (**Figure** **5**a; Figure S16, Supporting Information) and Mott–Schottky (MS) measurements (Figure 5b). Tauc plot analyses revealed that the optical band gaps for TTT‐TP‐COF, TTT‐DMTP‐COF, and TTT‐BMTP‐COF were 2.79, 2.64, and 2.58 eV, respectively (Figure 5b). The smaller band gap of TTT‐BMTP‐COF suggests a more efficiently fused conjugated structure than TTT‐TP‐COF and TTT‐DMTP‐COF. Mott–Schottky measurements indicated that the conduction band minimum of TTT‐BMTP‐COF was −0.84 V (vs RHE), which is more negative than TTT‐DMTP‐COF (−0.83 V) and TTT‐TP‐COF (−0.71 V), implying a stronger electron reduction capacity of TTT‐BMTP‐COF (Figure 5c).

To further elucidate the modulation effects of different alkoxy side chains on the catalytic mechanisms, DFT calculations were performed to identify the catalytical properties of the potential active sites involved in the ORR process. Both the lowest unoccupied molecular orbital (LUMO) and highest occupied molecular orbital (HOMO) were computed using VASP software. Analysis of the HOMO‐LUMO distributions across these COFs revealed distinct electronic characteristics influenced by alkoxy chain modifications (Figure 5d–f). TTT‐TP‐COF exhibited the most uniform orbital distribution, with the LUMO primarily localized on the benzene rings and the HOMO concentrated on the linkage groups. In contrast, TTT‐DMTP‐COF displayed a broader orbital distribution across multiple structural components, with the HOMO delocalized over the benzene rings, linkage groups, and alkoxy side chains. Notably, TTT‐BMTP‐COF showed the most pronounced side‐chain effects, characterized by an extended LUMO distribution on the alkoxy side chains and reduced HOMO–LUMO overlap, indicating favorable donor–acceptor interactions that facilitated charge transfer kinetics during electrocatalysis. Importantly, these side chain modifications primarily affect orbital spatial distribution rather than fundamental orbital patterns, indicating their potential as a tool for fine‐tuning electronic properties while maintaining core electronic structural features.

**Figure 6:**
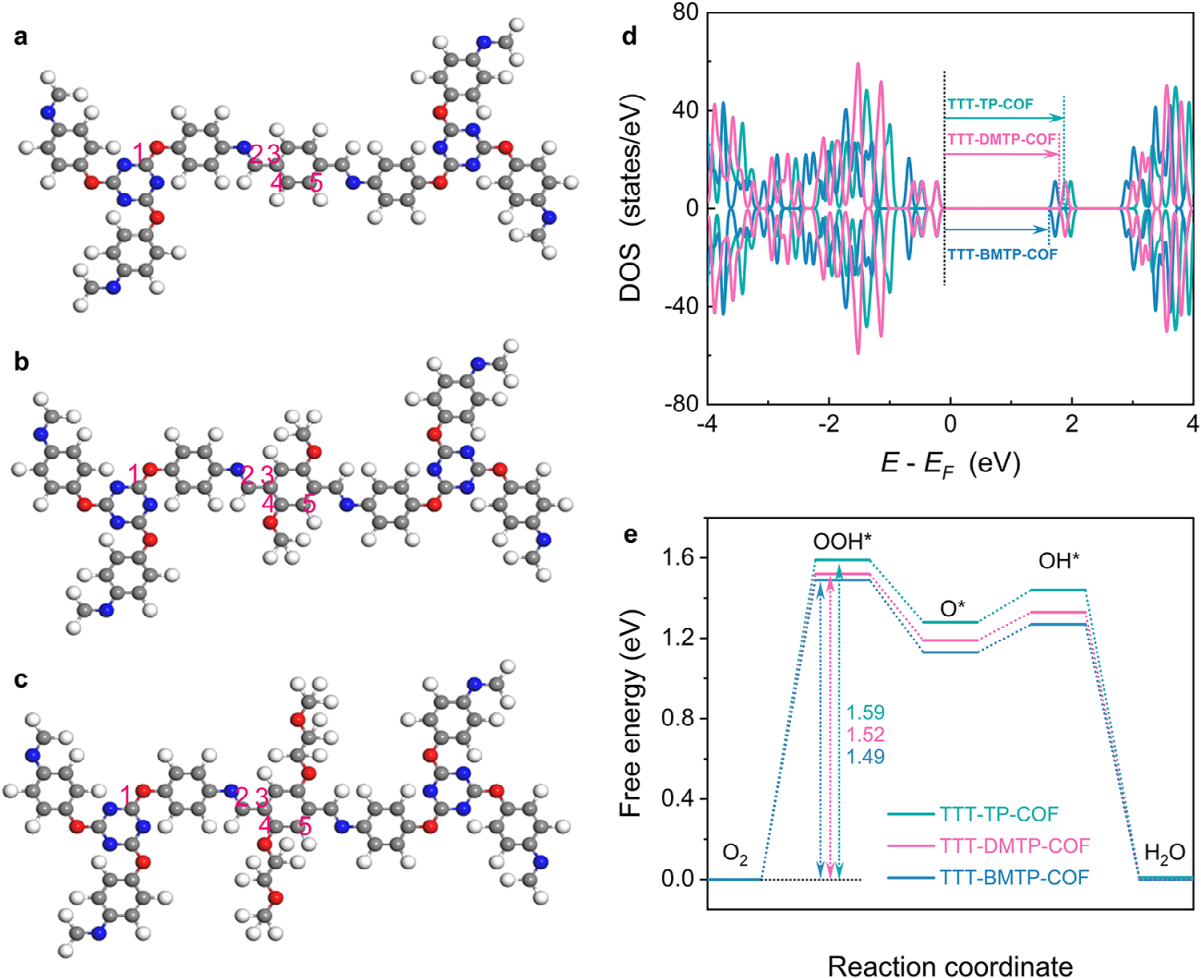
Optimized structures of a) TTT‐TP‐COF, b) TTT‐DMTP‐COF, and c) TTT‐BMTP‐COF based on calculations at the B3LYP/6‐31G (d,p) level. d) Density of states (DOS) and e) free energy diagrams for TTT‐TP‐COF, TTT‐DMTP‐COF, and TTT‐BMTP‐COF.

To further explore the influence of different alkoxy side chain structures on electronic properties and catalytic mechanisms, we calculated the electrostatic potential (ESP), total density of states, and free energy diagrams (**Figure** **6**a–e; Figure S17, Supporting Information) for TTT‐TP‐COF, TTT‐DMTP‐COF, and TTT‐BMTP‐COF. The ESP maps, shown in Figure S17 (Supporting Information), revealed a noticeable redistribution of electron density upon alkoxy modification in TTT‐BMTP‐COF and TTT‐DMTP‐COF compared to TTT‐TP‐COF. This redistribution supported the enhanced p–π conjugation and improved adsorption behaviour for ORR intermediates. Additionally, TTT‐BMTP‐COF exhibited the narrowest bandgap among the three COFs, suggesting improved charge transport properties induced by the alkoxy side chains (Figure 6d). For all potential active sites in TTT‐TP‐COF, TTT‐DMTP‐COF, and TTT‐BMTP‐COF, the Gibbs free energy changes for each elementary step in ORR were calculated using VASP software (Figures S18–S20, Supporting Information). TTT‐TP‐COF exhibited the lowest energy barrier at carbon sites near imine linkages (site 2 in Figure 6a), while TTT‐DMTP‐COF and TTT‐BMTP‐COF showed optimized catalytic activities at the carbon sites adjacent to the alkoxy chains (site 4 in Figure 6b,c), outperforming TTT‐TP‐COF (Figure 6e). For all three COFs, the rate‐determining step (RDS) was identified as the oxidation of an oxygen molecule to the adsorbed OOH intermediate, with energy barriers of 1.59, 1.52, and 1.49 eV for TTT‐TP‐COF, TTT‐DMTP‐COF, and TTT‐BMTP‐COF in Figure 6e and Figures S18–S20 (Supporting Information), respectively. In situ FT‐IR exhibited a readily identifiable OOH* peaks at ≈1226 cm^−1^, with increasing intensity caused by decreasing the applied potentials (Figure S21, Supporting Information). The OOH* peak of TTT‐BMTP‐COF was significantly intensified compared with that of other COF, indicating a more favorable reaction pathway toward ORR. These results demonstrated the activation effect of the alkoxy side chain modification. Furthermore, the energy barriers for the key steps at other potential sites were higher in Figures S18–S20 (Supporting Information), which further supported the beneficial role of alkoxy modification. The enhanced catalytic efficiency of TTT‐BMTP‐COF could be attributed to the alkoxy side chains, which not only facilitated charge transfer but also created additional active sites on the COFs, improving overall performance.

**Figure 7:**
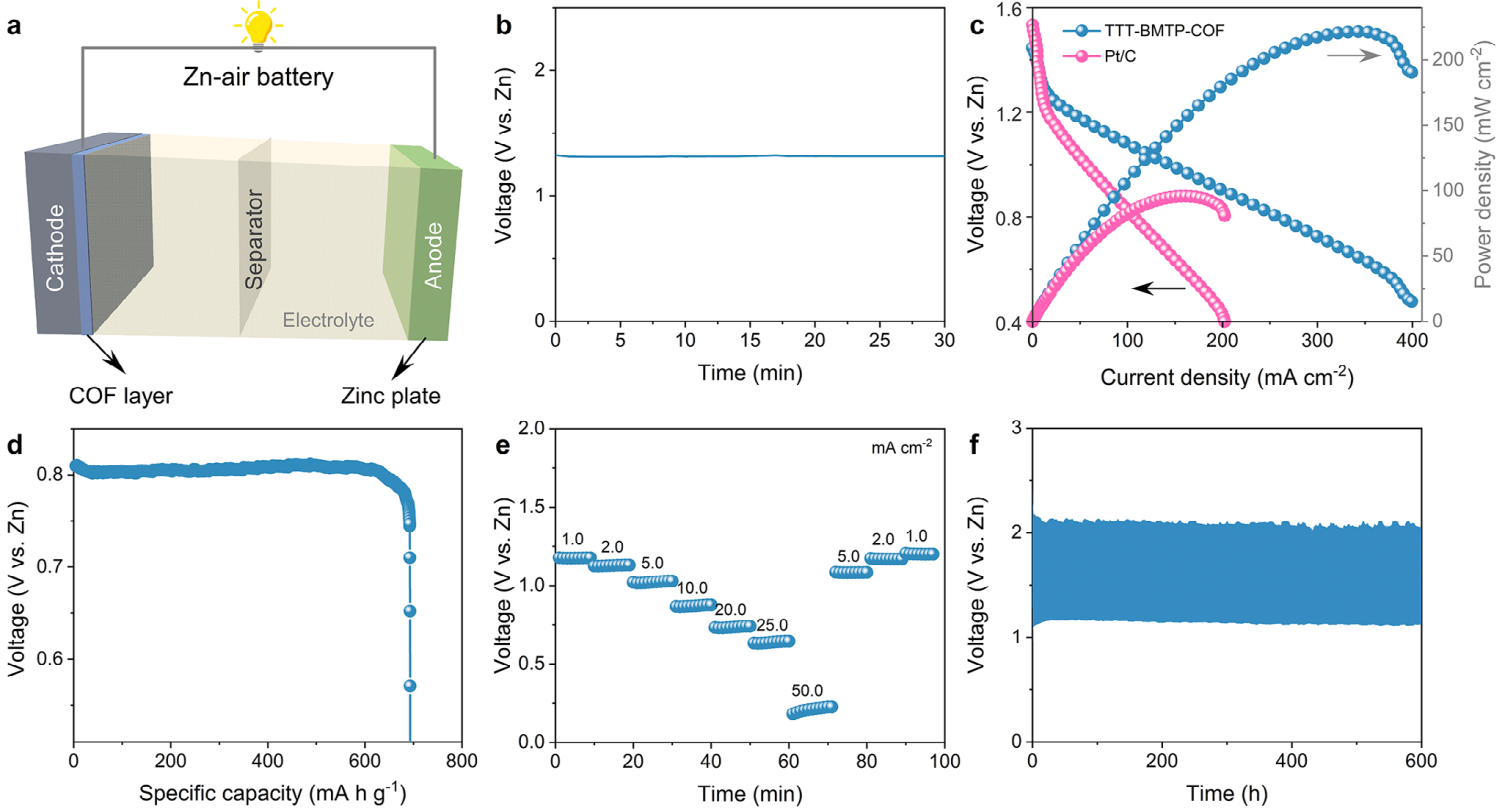
Performance of Zinc–air battery assembled with TTT‐BMTP‐COF. a) Diagram of an aqueous rechargeable ZABs, b) Open circuit voltage, c) Power density curves and charge–discharge polarization, d) Discharge curve at a constant current density of 20 mA cm^−2^, e) Rate discharging–charging curves at various current densities, and f) The cycling performance test of the ZAB fabricated with TTT‐BMTP‐COF measured at 5 mA cm^−2^.

TTT‐BMTP‐COF was assembled into zinc–air batteries (ZABs) to evaluate its practicality for the cathode part of ORR (**Figure** **7**a).^[^ ^58^, ^59^ ^]^ The TTT‐BMTP‐COF electrode exhibited an open‐circuit voltage of 1.32 V (Figure 7b) and a maximum power density of 221.6 mW cm^−2^ (Figure 7c), because of its favourable mass transfer properties. The specific capacity of the TTT‐BMTP‐COF electrode reached 692 mA h g^−1^ (Figure 7d), further validating the potential of COFs as effective catalysts in metal–air battery applications. Additionally, the TTT‐BMTP‐COF electrode demonstrated good discharge rate performance and stability, maintaining consistent performance with a continuous discharge at a current density of 5 mA cm^−2^ over 600 h (Figure 7e,f). For practical applications, we also investigated the performance of the catalyst in zinc–air batteries at a low temperature of −40 °C (Figures S22–S24, Supporting Information). Together, these results highlight the effects of the crucial side chains strategy for COF design, which allows the precise modulation of electronic properties for sustainable energy solution applications.

## 3 Conclusion

In summary, we demonstrated a potential application of metal‐free catalysts, COFs, which were prepared by modifying their pore wall surfaces with alkoxy side chains. These modifications enhanced key properties, including pore surface hydrophilicity, structural stability, and interactions with water molecules, while also tuning the electronic states of the catalytic active centers. Among the synthesized COFs, those with longer alkoxy side chains exhibited the highest catalytic activity, achieving a half‐wave potential of 0.77 V and a mass activity of 17.6 A g^−1^. Theoretical calculations suggested that this enhanced performance could be attributed to the increased binding affinity of water and *OOH intermediates to the carbon atoms adjacent to the alkoxy side chains. This study underscores the critical role of pore wall surface engineering for COF design and highlights the importance of side chains in modulating both the physical and electronic environments to achieve superior electrocatalytic performance. These insights provide valuable guidance to optimize COF‐based electrocatalysts, contributing to the development of more efficient energy conversion technologies.

## Conflict of Interest

The authors declare no conflict of interest.

## Author Contributions

Z.L., Z.W., and S.Z. contributed equally to this work. Z.L. and C.L. performed the primary experiments and data collection. Supporting experiments were conducted by S.Z, J.L., F.T., J.‐M.S., and W.‐Y.K. Z.W. and Z.L. was responsible for the theoretical calculations. This project was designed by C.L., Z.L., S.‐Y.L., and J.‐B.B. C.L., Z.L., S.‐Y.L., and J.‐B.B. wrote the manuscript, and all authors discussed the results and provided comments on the manuscript.

## Supporting information



Supporting Information

## Data Availability

The data that support the findings of this study are available from the corresponding author upon reasonable request.
