# Supplementary material for: Alkoxy Side Chain Engineering in Metal‐Free Covalent Organic Frameworks for Efficient Oxygen Reduction
Source: Adv Mater. 2025 Jul 4;37(38):2501603. doi: 10.1002/adma.202501603 (PMC12464629; doi:10.1002/adma.202501603)
Supplement: Supplementary file 1 — Supporting Information [file ADMA-37-2501603-s001.docx]

**Alkoxy Side Chain Engineering in Metal-free Covalent Organic Frameworks for Efficient Oxygen Reduction**

**Materials**

The KBr, o-DCB, n-BuOH, acetic acid, DMF, potassium hydroxide (KOH), Nafion resin (5 wt%), black carbon, tetrahydrofuran, acetone, methanol, and so on were obtained from Tokyo Chemical Industry Co., Ltd., Sigma Aldrich, and Samchun Chemical Co., Ltd. 4,4',4''-((1,3,5-Triazine-2,4,6-triyl)tris(oxy))trianiline (TTTOA), terephthalaldehyde (TPA), 2,5-dimethoxyterephthalaldehyde (DMTA), or 2,5-bis(2-methoxyethoxy)terephthalaldehyde (BMTA) were obtained from Jilin Zhongke Yanshen Technology Co., Ltd.

**Characterisation**

Fourier transform infrared (FT‑IR) spectra were recorded on a Perkin‑Elmer Spectrum 100 spectrometer using KBr pellets. UV-visible absorption spectra were recorded on a Cary 5000 spectrometer equipped with a D2 lamp. Solid state cross polarization magic-angle spinning nuclear magnetic resonance (ss CP-MAS NMR) spectra were recorded on an Agilent VNMRS 600 spectrometer at a spinning frequency of 10 kHz. Thermogravimetric analysis (TGA) measurements were conducted on an STA 8000 (Perkin-Elmer). The sample was heated from 50 to 800 °C at a heating rate of 10 °C min^−1^ under a nitrogen atmosphere. Powder X‑ray diffraction patterns were collected using a Rigaku D/Max 2500 rotating anode X‑ray powder diffractometer using Cu Kα radiation (λ = 1.5406 Å) operated at 1600 W (40 kV, 40 mA) power and equipped with a position sensitive detector with a 10.0 mm divergence height slit. Nitrogen sorption isotherms were recorded on a BELSORP‑max at 77 K. Prior to the measurements of the sorption isotherms, the samples were degassed for 24 h at 120 °C under high vacuum. The calculations of the pore size distribution were performed using the nonlocal density functional theory (NLDFT) adsorption model with cylindrical pores. High resolution transmission electron microscopy (HR-TEM) was performed with a JEOL JEM‐2100F equipped with a field emission gun operated at 200 kV. Field emission scanning electron microscopy (SEM) images were obtained using a Hitachi SU8220 Cold FE-SEM scanning electron microscope.

**Synthesis of COFs**

**Synthesis of TTT-TP-COF**: A Pyrex tube (16 mL) was charged with 4,4',4''-((1,3,5-triazine-2,4,6-triyl)tris(oxy))trianiline (TTTOA, 0.1 mmol), terephthalaldehyde (TPA, 0.15 mmol), 2.0 mL o-DCB/n-BuOH (v/v, 1:1), and 0.3 mL of 6 M aqueous acetic acid. The tubes were sonicated for two mins, flash frozen in liquid nitrogen bath and degassed by three freeze-pump-thaw cycles. The tubes were sealed off and heated at 120 °C for three days. The powder collected was washed with DMF, tetrahydrofuran, acetone and methanol several times, Soxhleted by tetrahydrofuran for 12 h, and then dried at 100 °C under vacuum for 12 h to obtain TTT-TP-COF (Yield: 89%).

**Synthesis of TTT-DMTP-COF**: A Pyrex tube (16 mL) was charged with 4,4',4''-((1,3,5-triazine-2,4,6-triyl)tris(oxy))trianiline (TTTOA, 0.1 mmol), 2,5-dimethoxyterephthalaldehyde (DMTA, 0.15 mmol), 2.0 mL o-DCB/n-BuOH (v/v, 1:1), and 0.3 mL of 6 M aqueous acetic acid. The tubes were sonicated for two mins, flash frozen in liquid nitrogen bath and degassed by three freeze-pump-thaw cycles. The tubes were sealed off and heated at 120 °C for three days. The powder collected was washed with DMF, tetrahydrofuran, acetone and methanol several times, Soxhleted by tetrahydrofuran for 12 h, and then dried at 100 °C under vacuum for 12 h to obtain TTT-DMTP-COF (Yield: 91%).

**Synthesis of TTT-BMTP-COF:** A Pyrex tube (16 mL) was charged with 4,4',4''-((1,3,5-triazine-2,4,6-triyl)tris(oxy))trianiline (TTTOA, 0.1 mmol), 2,5-bis(2-methoxyethoxy)terephthalaldehyde (BMTA, 0.15 mmol), 2.0 mL o-DCB/n-BuOH (v/v, 1:1), and 0.3 mL of 6 M aqueous acetic acid. The tubes were sonicated for two mins, flash frozen in liquid nitrogen bath and degassed by three freeze-pump-thaw cycles. The tubes were sealed off and heated at 120 °C for three days. The powder collected was washed with DMF, tetrahydrofuran, acetone and methanol several times, Soxhleted by tetrahydrofuran for 12 h, and then dried at 100 °C under vacuum for 12 h to obtain TTT-BMTP-COF (Yield: 83%).

**Stability measurements**

The COF samples were dispersed in water, various organic solvents, and aqueous NaOH and HCl solutions at room temperature for 24 hours to evaluate their chemical stability. After treatment, the samples were collected by simple filtration and sequentially washed with tetrahydrofuran, deionized water, and acetone. For samples treated under acidic conditions, additional washing was performed three times with aqueous ammonia. For those treated under basic conditions, the samples were washed three times with a small volume of acetic acid, followed by extensive rinsing with deionized water. Finally, all samples were washed with deionized water, acetone, and tetrahydrofuran, and then dried under vacuum at 70 °C for 12 hours.

**DFT calculations**

All DFT calculations were performed using the Vienna Ab-initio Simulation Package (VASP) with spin polarization.^1^ The exchange and correlation energies were approximated with the generalized gradient approximation (GGA) with the Perdew-Burke-Ernzerhof (PBE) functional.^2,3^ The Kohn-Sham one-electron states were extended in accordance with plane-wave basis sets with a cutoff energy of 520 eV.

A Gamma-only k-point mesh was applied for Brillouin zone (BZ) sampling in structure optimization and self-consistent field calculation of the freestanding ^1,2,3^ molecules with and without oxygenated adsorbates on different adsorption sites in each molecule in a 40*40*40 box. Van der Waals (vdW) dispersion energy correction was conducted using the DFT-D3 method.^4^ The energy and force convergence criteria were 1.0×10^–5^ eV and 0.01 eV/Å for structure relaxation, respectively. Calculated free energies were calibrated with entropic contributions using the VASPKIT software.^5^ The free energy diagrams for ORR were calculated with respect to the computational hydrogen electrode (CHE) model.^6^ The free energies of the ^1,2,3^ molecules with/without oxygenated adsorbates were calibrated according to the following equation:^5^

ΔG = ΔE + ΔZPE - TΔS - eU + ΔG_pH_

where ΔE, ΔZPE and - TΔS are the change in the single point energy, zero-point energy calibration and the entropic contribution to free energy. T = 298.15 K is taken as room temperature. U is the applied electrode potential. For pH calibration, we take ΔG_pH_ = -k_B_Tln[H^+^] = 0.0592 pH, where k_B_ is the Boltzmann constant.

In alkaline electrolyte, the electrocatalytic OER process (overall reaction: 4OH^-^ → O_2_ + 2H_2_O + 4e^-^) composed of the following four single-electron elementary reaction steps:^6-9^

OH^-^ + * → *OH + e^-^

*OH + OH^-^ → *O + H_2_O + e^-^

*O + OH^-^ → *OOH + e^-^

*OOH + OH^-^ → O_2_ + * + H_2_O + e^-^

And the electrocatalytic ORR process (overall reaction: O_2_ + 2H_2_O + 4e^-^ → 4OH^-^) in alkaline electrolyte composed of the following four single-electron elementary reaction steps:^10-13^

O_2_ + * + H_2_O + e^-^ → *OOH + OH^-^

*OOH + e^-^ → *O + OH^-^

*O + H_2_O + e^-^ → *OH + OH^-^

*OH + e^-^ → * + OH^-^

**Gaussian calculations**

Geometry optimization and self-consistent field calculations of all the COFs were conducted using the Gaussian 16 package^1^ and GaussView 6 software^2^. The COFs were optimized using the Density Functional Theory (DFT)/B3LYP method with 6-31g (d, p) basis set. The self-consistent field calculations were conducted using the same method with a larger 6-311+g (2d, p) basis set. Evaluation and visualization of electrostatic potential mapping was performed using a Multifunctional wavefunction analyzer (Multiwfn) ^3, 4^ and VMD programs, ^5^ respectively.

**Electrochemical measurements**

The working electrode was prepared using the following procedures: a rotating ring-disk electrode (RRDE) electrode was polished with 1.0 and 0.03 μm alumina slurry prior to each use, followed by rinsing thoroughly and sonication for 8 seconds with mixture of deionized water and ethanol to get a mirror-like surface. After that, TT-TP-COF, TTT-DMTP-COF and TTT-BMTP-COF (3 mg) and black carbon (3 mg) (Aldrich Chemical Inc.) were dispersed into deionized water (730 μL) and transferred for further sonication for 30 minutes, isopropyl alcohol (250 μL) and Nafion resin (5 wt %, 20 μL) were respectively introduced under sonication for another 1 h to get a uniform catalyst ink.^12^

All electrochemical experiments were carried out in a standard three-electrode cell system connected to an electrochemical workstation (Ivium, Netherlands), which was equipped with a rotating ring-disk electrode rotator (RRDE (5.61 mm in diameter), USA). Graphite rod and standard Ag/AgCl (3 M KCl) were used as the counter and reference electrodes, respectively. The potentials were converted to the values *versus* the RHE. The loading amounts for all catalysts were fixed at 0.3 mg cm^−2^.

For ORR measurement, a standard rotating ring-disk electrode loaded with various catalyst slurries was employed as the working electrode. All electrochemical tests were carried out at room temperature in N_2_ or O_2_-saturated 0.1 M aq. KOH electrolyte.^13^ The LSV test was performed at rotating speeds from 400 to 2025 rpm with a scan rate of 5 mV s^−1^.

The H_2_O_2_ yield, $\text{X}_{\text{H}_{\text{2}}\text{O}_{\text{2}}}$, electron transfer number *n*, and kinetic current density (j_k_) were calculated using the following equations:

$\text{X}_{\text{H}_{\text{2}}\text{O}_{\text{2}}}\text{=}\frac{\text{200}{\text{I}_{\text{r}}}/\text{N}}{\text{I}_{\text{d}}\text{+}{\text{I}_{\text{r}}}/\text{N}}$

$\text{n}\text{=}\frac{\text{4}\text{I}_{\text{d}}}{\text{I}_{\text{d}}\text{+}\frac{\text{I}_{\text{r}}}{\text{N}}}$

$j_{k}=\frac{j_{L}\times j}{j_{L-j}}$

where *I_d_* and *I_r_* are the disk and ring current, *N* is ring current collecting efficiency and equals 0.37, and j_L_ corresponds to the limiting current density.

The mass activity (MA) is a crucial parameter for quantitatively evaluating the intrinsic activity of an electrocatalyst. It is defined as the current density at a given potential (0.7 V vs. RHE), normalized by the catalyst loading. The MA is calculated using the following equation:

$MA=\frac{j}{m}$The turnover frequency (TOF) was evaluated by the following standard equation:

$$TOF=\frac{j\times A}{4 \times F \times n}$$

Where j (A cm^−2^) is the current density at a given potential (0.7 V vs. RHE), A is the surface area of the electrode, F stands for the Faraday constant (96500 C mol^−1^), the number of 4 represents 4 electrons mol^−1^ of O_2_, and n stands for the number of moles of C atoms in samples.

**Mott-Schottky measurands**

A total of 3 mg of catalyst was dispersed in 980 μL of ethanol, followed by the addition of 20 μL of 5 wt% Nafion solution. After sonication for 1 hour, 7 μL of the resulting suspension was drop-cast onto a glassy carbon electrode and dried to prepare the working electrode. A Ag/AgCl electrode served as the reference electrode, and a platinum wire was used as the counter electrode. Electrochemical measurements were carried out in 0.1 M Na_2_SO_4_solution using an electrochemical workstation. Mott–Schottky measurements were conducted in the potential range of –0.5 to 1 V at frequencies between 1000 Hz and 2000 Hz.

**In-situ FT-IR**

Nicolet iS50 FTIR spectrometer equipped with a narrow band MCT-A detector and an in-situ IR optical accessory (SPEC-I, Shanghai Yuanfang Tech) was used for in situ measurement. The incidence angle was approximately 60°. IR spectra were collected with polarized IR radiation at a spectral resolution of 2 cm^-1^. All spectra were presented in absorbance, defined as -log(R/R_0_), where R and R_0_ represent the sample and reference single-beam spectra, respectively. For potential control and current measurements, a CHI 760 electrochemistry workstation (CH Instruments, Inc.) was employed. Carbon rod and Ag/AgCl served as the counter electrode and reference electrode, respectively.

**Zn-air battery tests ^14, 15^**

The Zn-air battery tests were conducted with a homemade Zn-air cell. A well-polished Zn plate (0.5 mm thickness) was used as the anode. A 6 M KOH and 0.2 M ZnCl_2_ aqueous solution was selected as the electrolyte. Hydrophobic carbon paper (coating catalyst layer (1 mg cm^−2^) on the electrolyte-facing side and a gas-diffusion layer on the air-facing side) was used as the air cathode. All electrochemical experiments were carried out on a battery test system (LANHE CT2001A) at room temperature.

The specific capacity and power density were calculated with the following equation:

$\text{Specific capacity}\text{ =}\frac{\text{I × t}}{m_{Zn}}$

$\text{Power density}\text{ = }\frac{\text{ I × t × V }}{m_{Zn}}$

where *I* is the discharge current, *h* is the service hour, *m_Zn_* is the weight of consumed Zn and *V* is the discharge voltage.

The galvanostatic discharge-charge cycling curves were recorded at 10 mA cm^−2^ with 20 min per cycle (10 min for charge and 10 min for discharge).





**Figure S1.** FT IR of the COFs and building units. (a) TTT-TP-COF, (b) TTT-DMTP-COF, and (c) TTT-BMTP-COF.





**Figure S2**. (a) Full XPS spectra and (b) N 1s XPS spectra of TTT-TP-COF, TTT-DMTP-COF, and TTT-BMTP-COF.





**Figure S3**. ^13^C NMR spectra of (a) TTT-TP-COF, (b) TTT-DMTP-COF, and (c) TTT-BMTP-COF.





**Figure S4**. FE SEM images of (a) TTT-TP-COF, (b) TTT-DMTP-COF, and (c) TTT-BMTP-COF.





**Figure S5**. EDS Mapping images of TTT-TP-COF.





**Figure S6**. EDS Mapping images of TTT-DMTP-COF.





**Figure S7**. EDS Mapping images of TTT-BMTP-COF.





**Figure S8.** TGA curves of (a) TTT-TP-COF, (b) TTT-DMTP-COF, and (c) TTT-BMTP-COF.





**Figure S9.** Pore size curves of (a) TTT-TP-COF, (b) TTT-DMTP-COF, and (c) TTT-BMTP-COF.





**Figure S10**. PXRD patterns of (a) TTT-TP-COF, (b) TTT-DMTP-COF, and (c) TTT-BMTP-COF. Unit cells of AB-stacking model of (d) TTT-TP-COF, (e) TTT-DMTP-COF, and (f) TTT-BMTP-COF.





**Figure S11.** PXRD spectra of (a) TTT-TP-COF, (b) TTT-DMTP-COF, and (c) TTT-BMTP-COF under different conditions (as-synthesized: black: DMF: red; hexane: blue; water: green; HCl: purple; NaOH: yellow).





**Figure S12.** LSV curves of (a) TTT-TP-COF, (b) TTT-DMTP-COF, and (c) TTT-BMTP-COF at various rotation speeds (400 rpm-2025 rpm).





**Figure S13**. Limiting current density (j_L_) and kinetic current density (j_K_) of TTT-TP-COF, TTT-DMTP-COF, and TTT-BMTP-COF.





**Figure S14**. CV curves of (a) TTT-TP-COF, (b) TTT-DMTP-COF, and (c) TTT-BMTP-COF COF recorded in 0.1 M KOH solution at different scan rates (20, 40, 60, 80, 100 and 120 mV s^-1^).





**Figure S15**. Intrinsic activity of TTT-TP-COF and TTT-BMTP-COF calculated by normalizing current density by double-layer capacitance at given potentials (0.7 V and 0.6 V vs. RHE).





**Figure S16**. The electron absorption spectra of (a) TTT-TP-COF, (b) TTT-DMTP-COF, and (c) TTT-BMTP-COF.





**Figure S17**. Electrostatic potential (ESP) maps of (a) TTT-TP-COF, (b) TTT-DMTP-COF, and (c) TTT-BMTP-COF.





**Figure S18.** Free energy diagram of TTT-TP-COF calculated from different carbon sites.



**Figure S19**. Free energy diagram of TTT-DMTP-COF calculated from different carbon sites.



**Figure S20**. Free energy diagram of TTT-BMTP-COF calculated from different carbon sites.





**Figure S21**. In-situ FTIR spectra under applied potential range of 0.2 to −1.2 V vs Ag/AgCl: (a) TTT-TP-COF, (b) TTT-DMTP-COF, and (c) TTT-BMTP-COF.





**Figure S22.** Open circuit voltage of Zinc-air battery assembled with TTT-BMTP-COF at operated temperature of -40 °C.





**Figure S23**. Power density curves and charge–discharge polarization of Zinc-air battery assembled with TTT-BMTP-COF at operated temperature of -40 °C.

.





**Figure S24.** The cycling performance test of Zinc-air battery assembled with TTT-BMTP-COF at operated temperature of -40 °C.

.

**Table S1**. Element analysis of TTT-COFs

| Sample information | | C (%) | H (%) | N (%) |
| --- | --- | --- | --- | --- |
| TTT-TP-COFs | theoretical | 72.12 | 3.85 | 15.29 |
|  | observed | 71.86 | 4.99 | 15.01 |
| TTT-DMTP-COF | theoretical | 67.60 | 4.25 | 13.14 |
|  | observed | 66.78 | 5.13 | 12.97 |
| TTT-BMTP-COF | theoretical | 65.36 | 5.09 | 10.89 |
|  | observed | 64.75 | 6.14 | 9.66 |

**Table S2**. Atomic coordinates of TTT-TP-COF. *P*6, a = b = 37.89358 Å, c = 3.55002 Å, *α* = *β* = 90.00000°, *γ* = 120.00000°, *R*_wp =_ 5.42%, *R*_p =_ 4.02%

| N | 0.29707 | 0.66271 | 0.5 |
| --- | --- | --- | --- |
| C | 0.30075 | 0.63003 | 0.5 |
| O | 0.32347 | 0.73194 | 0.5 |
| C | 0.22892 | 0.58403 | 0.5 |
| C | 0.21846 | 0.6135 | 0.5 |
| C | 0.17896 | 0.6037 | 0.5 |
| C | 0.14915 | 0.56427 | 0.5 |
| C | 0.15931 | 0.53465 | 0.5 |
| C | 0.19885 | 0.54459 | 0.5 |
| N | 0.44487 | 0.55381 | 0.5 |
| C | 0.48061 | 0.56006 | 0.5 |
| C | 0.48817 | 0.52912 | 0.5 |
| C | 0.45822 | 0.48811 | 0.5 |
| C | 0.47389 | 0.46496 | 0.5 |
| H | 0.24235 | 0.64587 | 0.5 |
| H | 0.17097 | 0.62795 | 0.5 |
| H | 0.13525 | 0.50234 | 0.5 |
| H | 0.20696 | 0.52043 | 0.5 |
| H | 0.50654 | 0.5915 | 0.5 |
| H | 0.42485 | 0.47464 | 0.5 |
| H | 0.45399 | 0.43159 | 0.5 |

**Table S3**. Atomic coordinates of TTT-TP-COF. *P*6, a = b = 39.4423 Å, c = 3.4952 Å, *α* = *β* = 90.00000°, *γ* = 120.00000°, *R*_wp =_ 7.38%, *R*_p =_ 4.44%

| N | 0.29728 | 0.66271 | 0.5 |
| --- | --- | --- | --- |
| C | 0.30096 | 0.63023 | 0.5 |
| O | 0.32349 | 0.73153 | 0.5 |
| C | 0.22956 | 0.58449 | 0.5 |
| C | 0.21915 | 0.61379 | 0.5 |
| C | 0.1799 | 0.60405 | 0.5 |
| C | 0.15027 | 0.56484 | 0.5 |
| C | 0.16036 | 0.5354 | 0.5 |
| C | 0.19967 | 0.54528 | 0.5 |
| N | 0.44423 | 0.55454 | 0.5 |
| C | 0.47973 | 0.56073 | 0.5 |
| C | 0.4887 | 0.5287 | 0.5 |
| C | 0.45977 | 0.48868 | 0.5 |
| C | 0.4718 | 0.46058 | 0.5 |
| O | 0.47784 | 0.05792 | 0.5 |
| C | 0.43689 | 0.04421 | 0.5 |
| H | 0.2429 | 0.64597 | 0.5 |
| H | 0.17195 | 0.62814 | 0.5 |
| H | 0.13645 | 0.50328 | 0.5 |
| H | 0.20774 | 0.52127 | 0.5 |
| H | 0.50553 | 0.59197 | 0.5 |
| H | 0.44902 | 0.42806 | 0.5 |
| H | 0.42095 | 0.01528 | 0.65097 |
| H | 0.42596 | 0.04054 | 0.19807 |
| H | 0.43097 | 0.06581 | 0.65097 |

**Table S4**. Atomic coordinates of TTT-TP-COF. *P*6, a = b = 38.63637 Å, c = 3.54865 Å, *α* = *β* = 90.00000°, *γ* = 120.00000°, *R*_wp =_ 3.86%, *R*_p =_ 2.48%

| N | 0.29724 | 0.66264 | 0.5 |
| --- | --- | --- | --- |
| C | 0.30099 | 0.6302 | 0.5 |
| O | 0.32335 | 0.73146 | 0.5 |
| C | 0.2296 | 0.58435 | 0.5 |
| C | 0.21913 | 0.6136 | 0.5 |
| C | 0.17984 | 0.6038 | 0.5 |
| C | 0.15025 | 0.56457 | 0.5 |
| C | 0.1604 | 0.53516 | 0.5 |
| C | 0.19975 | 0.54511 | 0.5 |
| N | 0.44455 | 0.55483 | 0.5 |
| C | 0.48004 | 0.56098 | 0.5 |
| C | 0.48889 | 0.52885 | 0.5 |
| C | 0.45984 | 0.48887 | 0.5 |
| C | 0.47165 | 0.4606 | 0.5 |
| O | 0.47826 | 0.05821 | 0.5 |
| C | 0.43738 | 0.04476 | 0.5 |
| C | 0.07963 | 0.64851 | 0.5 |
| O | 0.39112 | 0.06659 | 0.5 |
| C | 0.10077 | 0.71188 | 0.5 |
| H | 0.24333 | 0.64647 | 0.5 |
| H | 0.17168 | 0.62836 | 0.5 |
| H | 0.13602 | 0.50236 | 0.5 |
| H | 0.20756 | 0.52029 | 0.5 |
| H | 0.50639 | 0.59286 | 0.5 |
| H | 0.44827 | 0.42745 | 0.5 |
| H | 0.42312 | 0.0259 | 0.76234 |
| H | 0.09848 | 0.65306 | 0.23771 |
| H | 0.70564 | 0.595 | 0.78237 |

**Reference**

1. G. Kresse, J. Furthmüller, *J. Phy. Rev. B* **1996**, *54*, 11169-11186.
2. J. P. Perdew, Burke, K., Burke, Ernzerhof, M. *Phys. Rev. Lett*. **1996**, *77*, 3865.
3. P. E. Blöchl, Projector augmented-wave method. *Phys. Rev. B* **1994**, *50*, 17953-17979.
4. S. Grimme, S. Ehrlich, L. Goerigk, *J. Comput. Chem*. **2021**, *32*, 1456-1465.
5. V. Wang, N. Xu, J. Liu, G. Tang, Geng, W. Geng, Comput. Phys. Commun. **2021**, *267,* 108033.
6. J. K. Nørskov, J. Rossmeisl, A. Logadottir, L. Lindqvist, J. R. Kitchin, T. Bligaard, H. *J.* Jónsson, *Phys. Chem. B* **2004**, *108,* 17886-17892.
7. Gaussian 16, Revision A.03, M. J. Frisch, G. W. Trucks, H. B. Schlegel, G. E. Scuseria, M. A. Robb, J. R. Cheeseman, G. Scalmani, V. Barone, G. A. Petersson, H. Nakatsuji, X. Li, M. Caricato, A. V. Marenich, J. Bloino, B. G. Janesko, R. Gomperts, B. Mennucci, H. P. Hratchian, J. V. Ortiz, A. F. Izmaylov, J. L. Sonnenberg, D. Williams-Young, F. Ding, F. Lipparini, F. Egidi, J. Goings, B. Peng, A. Petrone, T. Henderson, D. Ranasinghe, V. G. Zakrzewski, J. Gao, N. Rega, G. Zheng, W. Liang, M. Hada, M. Ehara, K. Toyota, R. Fukuda, J. Hasegawa, M. Ishida, T. Nakajima, Y. Honda, O. Kitao, H. Nakai, T. Vreven, K. Throssell, J. A. Montgomery, Jr., J. E. Peralta, F. Ogliaro, M. J. Bearpark, J. J. Heyd, E. N. Brothers, K. N. Kudin, V. N. Staroverov, T. A. Keith, R. Kobayashi, J. Normand, K. Raghavachari, A. P. Rendell, J. C. Burant, S. S. Iyengar, J. Tomasi, M. Cossi, J. M. Millam, M. Klene, C. Adamo, R. Cammi, J. W. Ochterski, R. L. Martin, K. Morokuma, O. Farkas, J. B. Foresman, and D. J. Fox, Gaussian, Inc., Wallingford CT, 2016.
8. GaussView, Version 6.0.16, R. Dennington, T. A. Keith, J. M. Millam, Semichem Inc., S. Mission, KS, 2016.
9. T. Lu, F. Chen, *J. Comput. Chem*, **2012**, *33*, 580-592.
10. J. Zhang, T. Lu. *Phys. Chem. Chem. Phys.*, **2021**, *23*, 20323-20328.
11. W. Humphrey, A. Dalke, K. Schulten. *J. Mol. Graph*., **1996**, *14*, 33-38.
12. M. Liu, J. Zhang, H. Su, Y. Jiang, W. Zhou, C. Yang, S. Bo, J. Pan, Q. Liu. *Nat. Commun*. **2024**, *15*, 1675.
13. X. Yi, H. Yang, X. Yang, X. Li, C. Yan, J. Zhang, L. Chen, J. Dong, J. Qin, G. Zhang, J. Wang, W. Li, Z. Zhou, G. Wu, X. Li. *Adv. Funct. Mater*. **2024**, *34*, 2309728.
14. J. C., H. Li, C. Fan, Q. Meng, Y. Tang, X. Qiu, G. Fu, T. Ma, *Adv. Mater.* **2020**, *32*, 2003134.
15. S. Ji, Y. Mou, H. Liu, X. Lu, Y. Zhang, C. Guo, K. Sun, D. Liu, J. H. Horton, C. Wang, Y. Wang, Z. Li, *Adv. Mater*. **2024**, *36*, 2410121.
